# Multilocus sequence typing of clinical *Burkholderia pseudomallei* isolates from Cambodia

**DOI:** 10.1371/journal.pntd.0012652

**Published:** 2024-11-14

**Authors:** Emmanuel Gyamfi, Gauthier Delvallez, Sokleaph Cheng, Soda Meng, Kimyeun Oeurn, Chanchakriya Sam, Alexandra Kerleguer, Bertrand Guillard, Anne-Laure Bañuls, Mallorie Hide

**Affiliations:** 1 Medical Biology Laboratory, Institut Pasteur du Cambodge, Phnom Penh, Cambodia; 2 LMI Drug Resistance In Southeast Asia, Institut Pasteur du Cambodge, Phnom Penh, Cambodia; 3 MIVEGEC, Montpellier University, CNRS, IRD, Montpellier, France; University of Florida, UNITED STATES OF AMERICA

## Abstract

Melioidosis is a neglected tropical disease caused by *Burkholderia pseudomallei*, endemic to Southeast Asia and Northern Australia. Despite its increasing global public health and clinical significance, the molecular epidemiology of melioidosis and genetic diversity of *B*. *pseudomallei* in Cambodia remains poorly understood. This study aims to elucidate the genetic diversity and antibiotic susceptibility profiles of *B*. *pseudomallei* isolates responsible for melioidosis in humans. For this purpose, 14 clinical isolates cryopreserved at the Medical Biology Laboratory at Institut Pasteur du Cambodge from 2016 to 2020 were subjected to antimicrobial susceptibility testing and Multilocus Sequence Typing (MLST). Phenotypic testing revealed that 92.86% (13/14) of the isolates were sensitive to all tested antibiotics, while one isolate exhibited resistance to trimethoprim-sulfamethoxazole. MLST analysis resolved our isolates into 14 unique Sequence Types (STs), including 10 previously documented in Southeast Asia. Notably, ST1858, ST2064, ST2065, and ST2066 were identified as novel STs, while ST54, ST99, ST211, and ST1359 were reported in Cambodia for the first time in this study. Comparing our MLST data with available sequences on PubMLST (n = 165), our study unveiled a high genetic diversity of *B*. *pseudomallei* in Cambodia. The identified STs were closely associated with isolates from other Southeast Asian countries, particularly Thailand, Vietnam, and Malaysia. In conclusion, this study provided insight into the genetic diversity among *B*. *pseudomallei* clinical isolates in Cambodia and their close genetic association with Southeast Asian isolates. To further our understanding, a One Health approach, incorporating human, environmental (mainly soil), and animal compartments, is essential to decipher the epidemiology of *B*. *pseudomallei* in Cambodia.

## Author summary

Melioidosis, caused by the soil-dwelling bacterium *Burkholderia pseudomallei*, is commonly found in regions of Southeast Asia and northern Australia. Despite appropriate antibiotic treatment, it remains a potentially fatal disease. The clinical manifestations are varied, and the lack of laboratory support in Cambodia leads to underreporting. Limited genetic data are available for *B*. *pseudomallei* in Cambodia; this study provides new insights into the genetic characteristics of this bacteria and antibiotic susceptibility in the country, benefiting public health and safety. Using multi-locus sequence typing (MLST), we confirm that Cambodian clinical isolates are closely related to the Southeast Asian cluster, particularly those from Thailand, Vietnam, and Malaysia, reflecting the regional endemicity. This study also reveals new sequence types and antibiotic resistance to the trimethoprim/sulfamethoxazole combination. To further our understanding, a One Health approach, incorporating human, environmental (mainly soil), and animal compartments, is essential to decipher the epidemiology of *B*. *pseudomallei* in Cambodia.

## Introduction

The *Burkholderia* genus is commonly separated into two major phylogenetic groups: the *Burkholderia pseudomallei* complex (BPC), consisting of *B*. *pseudomallei* and its most closely related phylogenetic relatives, and the *Burkholderia cepacia* complex (BCC) [1,2]. BPC includes *B*. *pseudomallei*, an environmental Gram-negative saprophytic bacteria, responsible for melioidosis. *B*. *pseudomallei* is transmitted to humans by contaminated soil and surface water (percutaneous inoculation, inhalation, or ingestion) and person-to-person or zoonotic transmissions are extremely rare [3]. Climate factors such as weather event, flooding and other environmental disturbance have a huge impact on melioidosis epidemiology [4].

Most *Burkholderia* species contain a modified lipopolysaccharide that causes intrinsic polymyxin resistance (reduced drug penetration—restrictive porin proteins). Efflux pumps of the resistance nodulation cell division family are major players in *Burkholderia* multidrug resistance [5]. *B*. *pseudomallei* exhibits resistance to a wide range of antibiotics, including penicillins, first- and second-generation cephalosporins, macrolides, colistin and aminoglycosides. Resistant strains to ceftazidime and amoxicillin-clavulanic acid have been reported, and new resistant strains continue to emerge [6].

This disease is considered as endemic in Southeast Asia (SEA) but its molecular epidemiology remains poorly documented in Cambodia partly due to the lack of clinician awareness and limited diagnostic microbiology capacity [7]. Since 2005, human melioidosis cases began to be regularly identified in-country [8,9] even though both animal and exported human cases predate the first endemic case report [10–12]. Since then, several studies reported *B*. *pseudomallei* in Cambodia, confirming its continuing endemicity [7,13–17]. Unfortunately, without systematic surveillance and high throughput research, the real burden of the disease in the country appears to be underestimated [7]. In addition, data on the epidemiology of melioidosis in Cambodia are limited with only a few studies in the scientific literature. For several years, there has been a steady increase in the number of culture-confirmed cases and the report provided by the Cambodia Training Event for Awareness of Melioidosis (C-TEAM) recorded 2592 confirmed melioidosis cases in Cambodia between 2005 and 2017 [7]. The C-TEAM is a national One Health training event held in Phnom Penh from October 17 to 19, 2017. Its goal was to enhance the awareness of melioidosis among clinical, laboratory, and public health professionals in Cambodia. The treatment consists of ceftazidime during the initial phase, followed by the trimethoprim/sulfamethoxazole combination (SXT) during the eradication phase [7]. Since 2016, the annual number of confirmed melioidosis cases in Cambodia is greater than 450 cases. In this country, according to the C-TEAM report, the majority of cases are children (>60%) with head and neck infections likely due to overrepresentation of cases from three children’s hospitals in the C-TEAM survey. In contrast, adults most commonly present with pneumonia and/or sepsis [7] with risk factors such as diabetes mellitus, renal diseases, chronic lung disease, thalassemia, excessive alcohol consumption, male gender, immunosuppression and occupational exposure [18].

MultiLocus Sequence Typing (MLST) is a molecular technique used to characterize isolates for epidemiological studies, it is based on allelic variations in seven housekeeping genes that are expected to be neutral and it is the most widespread genotyping methodology for bacterial pathogens [19]. Few genetic data are available for *B*. *pseudomallei* in Cambodia on PubMLST database but their analysis has not been published. In this context, our objective was to describe *B*. *pseudomallei* by exploring the genetic diversity and resistance status of isolates responsible for melioidosis, isolated and cryopreserved at the Medical Biology Laboratory, Institut Pasteur du Cambodge (MBL-IPC), Phnom Penh, Cambodia from 2016 to 2020. All the bacterial isolates were subcultured to confirm their identification using Bruker Maldi Biotyper before to characterize their phenotypic resistance and their sequence type (ST) using the Multilocus Sequence Typing (MLST). Their MLST profiles were compared with all the MLST profiles available on PubMLST for Cambodia (n = 165).

## Methods

### Bacterial isolates and identification

Fourteen *B*. *pseudomallei* strains, isolated and cryoconserved at MBL-IPC between 2016 and 2020 from clinical samples provided by different hospitals in Phnom Penh for routine diagnostic microbiology procedures, were included in this retrospective study. These clinical samples were collected from adults living near Phnom Penh who were hospitalized for melioidosis. All isolates were first identified as *B*. *pseudomallei* using API 20 NE (bioMérieux, Marcy l’Etoile, France). Prior to genetic analysis, identification of all isolates was subsequently confirmed as *B*. *pseudomallei* isolates by matrix-assisted laser-desorption/ionization time-of-flight mass spectrometry (MALDI-TOF MS) using Bruker Biotyper with MBT Security-Relevant (SR) Library.

### Antibiotic susceptibility testing

Antibiotic susceptibility testing (AST) was performed for the 14 *B*. *pseudomallei* isolates by disc diffusion method in accordance with the European Committee on Antimicrobial Susceptibility Testing (EUCAST 2023) guidelines [20] for the following antibiotics (Bio-Rad): amoxicillin-clavulanic acid (20–10 μg), ceftazidime (10 μg), imipenem (10 μg), trimethoprim-sulfamethoxazole (1.25–23.75 μg), and tetracycline (30 μg). In case of reduced diameter, the result was verified by determination of minimum inhibitory concentration (MIC) using E-test (Biomerieux) as per the manufacturer’s instruction. *Escherichia coli* ATCC 25922, a recommended reference strain for AST, was used as a control.

### DNA extraction

Subcultures and DNA extractions were conducted under enhanced BSL2+ conditions. These enhanced conditions include working strictly within the BSCII and employing PPE similar to that used in BSL3 settings, such as Tyvek suits, double gloving, and FFP3 masks. Genomic DNA was extracted from the *B*. *pseudomallei* subcultures using the heat treatment with slight modifications [21]. Overnight subcultures were placed into 100 microliters of sterile water, boiled in a water bath for 5 minutes, transferred into ice for 2 minutes then centrifuged at 12,000 ×g for 10 minutes at 4°C. The supernatant was transferred into a new tube and stored at -20°C.

### Multilocus sequence typing & analysis

*B*. *pseudomallei* isolates were amplified for seven housekeeping genes (*ace*, *gltB*, *gmhD*, *lepA*, *lipA*, *narK*, *ndh*) [19] and sequenced by Macrogen, Korea for Capillary Electrophoresis sequencing. Sequences were cleaned and aligned using MEGA X [22] for phylogenetic reconstruction and submitted to the PUBMLST database for registration (http://pubmlst.org/bpseudomallei/). iTOL version 6 software was used for tree editing [23] (http://itol.embl.de/). Genetic relatedness of the isolates was analysed using the goeBURST algorithm and allowed to infer patterns of evolutionary descent among clusters of *B*. *pseudomallei* strains from MLST data [24].

## Results

### Identification & Phenotypic resistance

All the isolates were confirmed as *B*. *pseudomallei* by MALDI-TOF. Regarding antibiotic susceptibility testing, 13 isolates were susceptible to all tested antibiotics whereas one isolate (MBL17-12) showed resistance to trimethoprim-sulfamethoxazole with a MIC greater than 32 mg/l. One isolate (MBL17-09) showed reduced inhibition zones for both amoxicillin-clavulanic acid and ceftazidime. However, the MICs estimated by E-test remained within the susceptible range, at 6 mg/l and 3 mg/l, respectively.

### Multilocus sequence typing

We obtained complete allelic profiles (seven loci) for the 14 *B*. *pseudomallei* strains. These MLST profiles have been submitted to PUBMLST (id 6726–6739), bringing the total of *B*. *pseudomallei* isolates from Cambodia to 179. The 179 MLST profiles are available on PubMLST (“search or browse database”, select: “country = Cambodia”) (S2 Table).

Fourteen different STs were obtained, including four novel STs assigned as ST1858, ST2064, ST2065 and ST2066 by PubMLST curator as shown in Table 1. The numbers of alleles vary between 2 and 5, *ndh* and *gmhD/nark*, being respectively the less and the most polymorphic loci (Table 2). Upon querying the *B*. *pseudomallei* PubMLST database, we found that the remaining 10 STs (34, 54, 99, 178, 196, 211, 381, 438, 531, 1359) have already been described, essentially in SEA (Indonesia, Cambodia, Lao People’s Democratic Republic, Malaysia, Philippines, Thailand, Vietnam and Singapore) but also in other Asian countries (Bangladesh, China). Concerning Cambodia, six STs among 14 have been previously reported in Cambodia (ST34, 178, 196, 381, 438, 531) among the 107 STs described in Cambodia on *B*. *pseudomallei* PubMLST database within the 165 Cambodian isolates (1996–2011). All six have been previously isolated from human patients in Cambodia in the period 2007–2009 and have as well been detected in Thailand, Vietnam and Malaysia (Table 1.). The remaining eight STs were described for the first time in Cambodia in this study (ST54, 99, 211, 1359, 1858, 2064, 2065, 2066). Considering the entire PubMLST database, 179 isolates from Cambodia corresponded to 121 STs, resulting in an overall diversity of 0.68 STs per isolate.

**Table 1:** MLST profiles for the 14 *B*. *pseudomallei* isolates.

Isolate	PubMLST ID	year	ace	gltB	gmhD	lepA	lipA	narK	ndh	ST	ST geographical distribution* & Source (number of isolates)
MBL16-01	6726	2016	1	4	12	1	6	4	1	2064	New, KH/Human (1)
MBL16-02	6727	2016	1	4	12	1	1	4	1	34	KH/Human (5); MY/Human (2); TH/Human (10)/Env (1)
MBL16-03	6728	2016	1	2	2	1	1	3	1	381	KH/Human (2); TH/Env (1); VN/Human (3)
MBL16-04	6729	2016	3	4	3	3	1	4	1	1858	New, KH/Human (1)
MBL16-05	6730	2016	3	1	3	1	1	4	1	211	CN/Human (4); BD/Human (1); KH/Human (1); TH/Human (2); VN/Human (5)
MBL16-06	6731	2016	3	1	3	3	1	2	1	54	KH/Human (1); ID/Animal (1); LA/Env (2); MY/Human (38); SG/Animal (1); TH/Human (20)/Env (14)
MBL17-07	6732	2017	1	2	3	2	1	4	3	2065	New, KH/Human (1)
MBL17-08	6733	2017	1	1	3	4	5	22	1	178	KH/Human (5); TH/Human (3)/Env (1)
MBL17-09	6734	2017	1	4	2	3	1	4	1	531	KH/Human (2); TH/Human (3)
MBL17-10	6735	2017	1	1	4	1	1	4	1	99	KH/Human (1); MY/Human (1); PH/Human (1); TH/Human (3)/Env (3)/Animal (2)
MBL17-11	6736	2017	3	4	11	4	5	4	1	1359	KH/Human (1); MY/Human (7); TH/Human (4)
MBL17-12	6737	2017	3	1	11	1	1	22	1	196	KH/Human (4); TH/Env (1); VN/Env (1)
MBL19-13	6738	2019	4	1	11	2	1	1	1	2066	New, KH/Human (1)
MBL20-14	6739	2020	1	1	2	3	1	2	1	438	KH/Human (2); MY/Human (5)

*From PubMLST database. « KH/Human (1) » means that the corresponding ST is described for the first time in human in Cambodia. « New » means that the corresponding ST is a new ST recorded thanks to this study.

Abbreviations: BD, Bangladesh; CN, China; Env, environmental source; ID, Indonesia; KH, Cambodia; LA, Lao People’s Democratic Republic; MY, Malaysia; PH, Philippines; SG, Singapore; ST, Sequence Type; TH, Thailand; VN, Vietnam. The 14 MLST profiles are available on PubMLST database.

**Table 2:** Allele diversity for the 14 *B*. *pseudomallei* isolates.

Locus	No, of alleles	Alleles
*ace*	3	1, 3, 4
*gltB*	3	1, 2, 4
*gmhD*	5	2, 3, 4, 11, 12
*lepA*	4	1, 2, 3, 4
*lipA*	3	1, 5, 6
*narK*	5	1, 2, 3, 4, 22
*ndh*	2	1, 3

**Fig 1:**
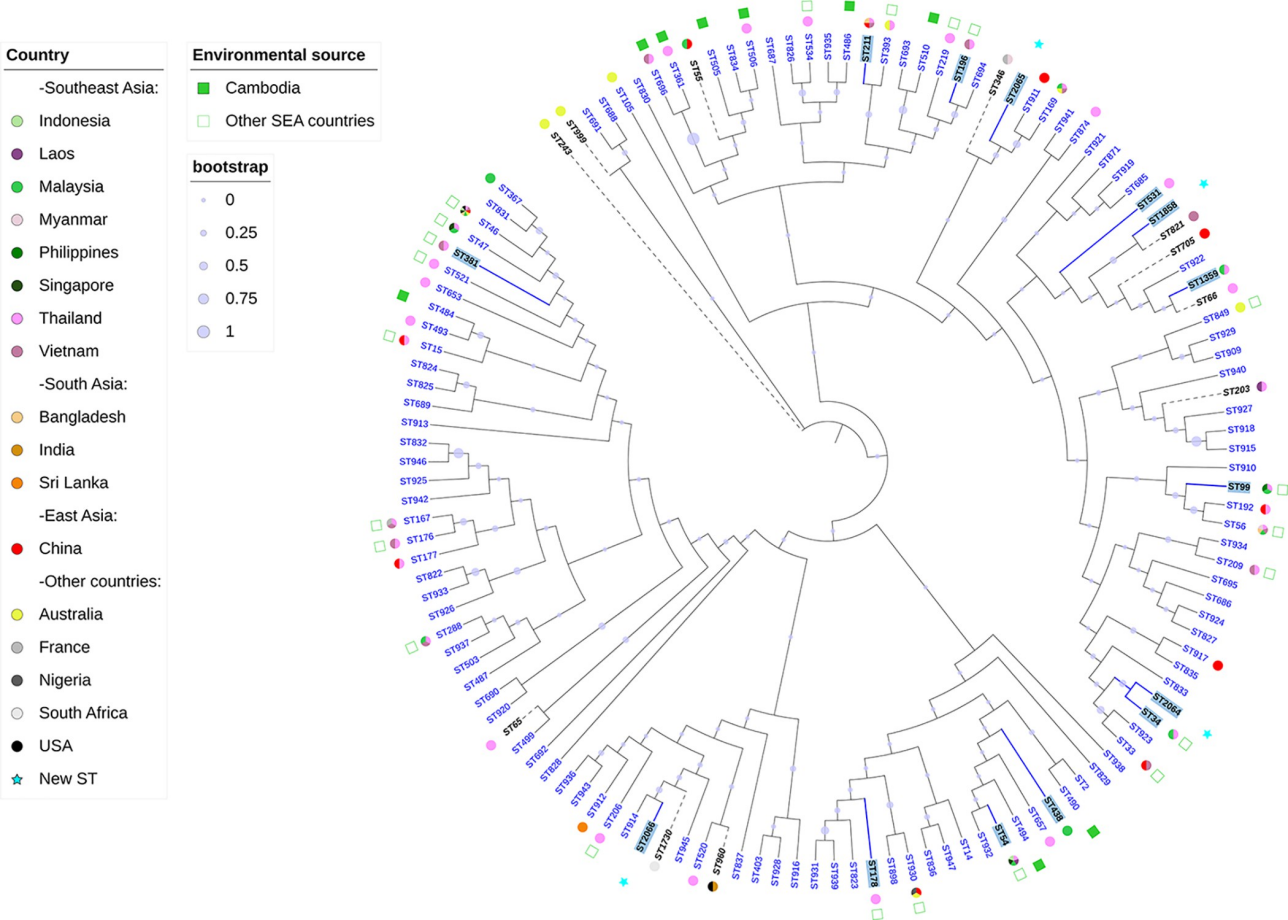
Maximum Likelihood tree based on PubMLST data. The 14 *B*. *pseudomallei* included in this study are highlighted in blue (label and branch). 165 MLST profiles from Cambodia are written in blue and additional MLST profiles are represented by the dotted lines. SEA, Southeast Asia; USA, Unites States of America.

Our MLST sequences were aligned with the 165 MLST sequences available on PubMLST for Cambodia, nine additional profiles sharing six among seven loci with the novel ST1858/ST2065/ST2066 and two profiles from Australia (Outgroup) (Fig 1 and S1 Table). It should be noted that the new ST2064 shares six of the seven alleles with only two STs (ST34 and ST921) already included in MLST 165 from Cambodia. Fig 1 presents the Maximum Likelihood tree, the 14 isolates sequenced in this study are highlighted in blue (label and branch) and the geographic origin of the STs is represented on the pie chart. Our 14 *B*. *pseudomallei* isolated between 2016 and 2020 showed an important genetic diversity, are dispersed in different clusters and are mixed with the other Cambodian isolates. No structuration was revealed comparing the isolation year. The patient’s data were not available and unable us to explore geographic data.

**Fig 2:**
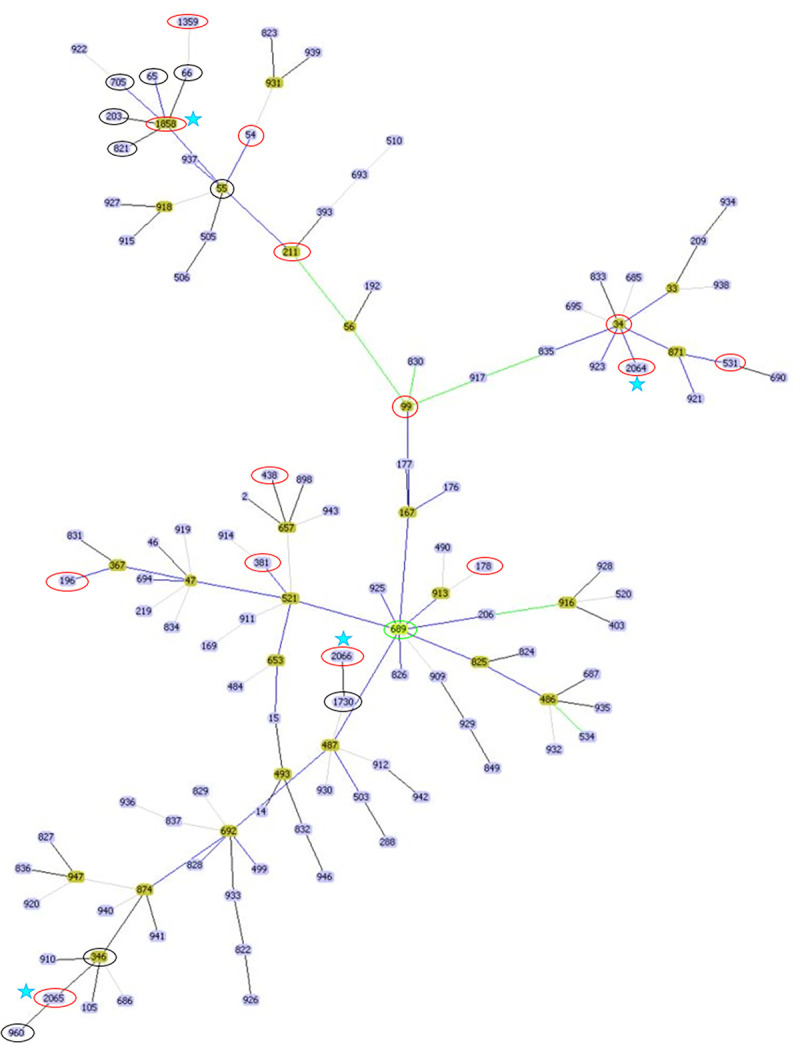
Population snapshot of *B*. *pseudomallei* (goeBURST). The 14 isolates included in this study are circled in red, the nine additional STs (harboring six loci among seven in common with the new STs identified in this study) are circled in black and the four novel STs are indicated by a blue star. ST689 is identified as the predicted founder and is circled in green.

The goeBURST tool was used to infer patterns of evolutionary descent among clusters of *B*. *pseudomallei* strains from MLST data. The dataset comprised the 121 STs identified in Cambodia as well as nine additional STs close to the new STs identified in this study (ST1858, ST2065, ST2066) which harbor six MLST loci in common with these new STs. The goeBURST diagram allowed clustering 123 isolates among 130 with the double locus variants (DLV) option (Fig 2). Seven STs could not be related to other isolates (ST361, 494, 688, 691, 696, 924, and 945). Five of them were represented by a single isolate whereas ST361 corresponded to two isolates from Cambodia and Thailand and ST696 corresponded to 30 isolates from Cambodia (n = 1), Thailand (n = 32) and Vietnam (n = 1). ST689 was identified as the predicted founder regardless the selected option (single locus variants (SLV) or double locus variants (DLV)). ST689 has been described in 2007 and 2008 for 2 isolates from humans in Cambodia. The four novel STs (ST1858, 2064, 2065, and 2066) were linked to distinct complexes, respectively, with ST55, ST34, ST346, and ST487 as founders (Fig 2).

## Discussion

Limited genetic data are available for *B*. *pseudomallei* in Cambodia and this study provides new insights into the genetic characteristics of this bacteria and antibiotic susceptibility in the country, benefiting public health and safety. We investigated the genetic diversity of *B*. *pseudomallei* isolated from humans in Cambodia between 2016 and 2020, noting the lack of additional MLST profiles submitted to PubMLST since 2010. MLST analysis revealed 14 unique STs, including 8 STs described for the first time in Cambodia. It is widely accepted that *B*. *pseudomallei* was likely introduced into SEA from Australia through a single transmission event a during a recent glacial period [25,26]. Of the 14 STs identified, four are novel, while the remaining ten have been previously reported in SEA in clinical, animal, and/or environmental samples. Among the previously reported STs, all have been detected in human samples, with six also found in environmental samples and two in animal samples. Phylogenetic analysis of the 14 STs from Cambodia indicates a close association with Southeast Asian isolates, particularly those from Thailand (9/10), Vietnam (3/10), and Malaysia (5/10). Similar findings were reported in Malaysia, where authors identified an association with STs from Thailand [27]. Using both maximum likelihood approach and eBURST algorithm, the 14 STs were dispersed across different clusters, showing a closer genetic relationship to STs from Southeast Asia than to each other. Transmission among countries bordered by the Mekong River (Thailand, Laos, Cambodia, and Vietnam) has previously been highlighted and is in agreement with our findings [25].

This study, focusing on patients from hospitals in Phnom Penh, revealed a notably high overall genetic diversity (0.68 STs/isolate) compared to previous MLST analyses of *B*. *pseudomallei* from neighboring Southeast Asian countries. For instance, a study from Malaysia identified 29 sequence types (STs) among 84 isolates (0.35 STs/isolate), predominantly associated with Southeast Asian strains, particularly those from Thailand [27]. The significant genetic variability observed in this study aligns with the extensive recombination and horizontal gene transfer events previously described within this species [26]. Additionally, this variability may be explained by the time range (2016–2020) and the spatial distribution, as the locations of the patients are unknown.

Additionally, Vesaratchavest et al. (2006) demonstrated that clinical isolates exhibit less genetic diversity compared to environmental ones [28]. Unfortunately, in Cambodia, *B*. *pseudomallei* has only been detected in paddy field in Siem Reap without genetic analysis [17] and among the 179 samples available for Cambodia on PubMLST, only 9 originate from environmental sources, representing 9 distinct STs and isolated in 2005–2006 (green squares on Fig 1). These nine STs are distributed within different clusters, with two (ST490 and ST506) also isolated from humans. Additionally, among the 170 remaining Cambodian STs, twenty-two (highlighted with a green square outline in Fig 1) have been isolated from environmental sources in other Southeast Asian countries, particularly Thailand. Among our 14 STs, six out of the 10 previously described have been identified in the environment in Thailand (6/6), Vietnam (1/6), and Laos (1/6) (Table 1). These analyses are in agreement with the circulation of *B*. *pseudomallei* among countries bordered by the Mekong River [25]. Recently, a study in Thailand highlighted the persistence of *B*. *pseudomallei* in the environment as a pivotal element facilitating the bacterial spread [29]. The authors also revealed a clustering of both clinical and environmental isolates indicating their core genetic similarities and their potential epidemiological link. The burden of *B*. *pseudomallei* in Cambodia and across SEA is of a great concern due to its wide distribution in the community as an environmental etiological agent. Consequently, exploring the genetic diversity among both environmental and human isolates within the same geographical area is crucial for a better understanding of the disease’s circulation in Cambodia. In this context, our team has recently isolated *B*. *pseudomallei* in both soil and water in Kandal Province and whole genome sequencing is currently ongoing (personal communication).

Among the eight sequence types (STs) described for the first time in Cambodia in this study, four (ST54, 99, 211, and 1359) have previously been reported in SEA in association with human samples. Additionally, ST54, reported in Indonesia, Laos, Malaysia, Singapore and Thailand, along with ST99, found in Malaysia, the Philippines and Thailand, have both been detected in animal and environmental samples (Table 1). The remaining four STs (ST1858, 2064, 2065, and 2066) are novel, being exclusive to Cambodia thus far. According to the goeBURST snapshot, all four novel STs are linked to distinct subclonal complexes, with predicted founders isolated in various Asian countries (Cambodia, China, Malaysia, Myanmar, and Thailand). This observation is in agreement with repeated reintroductions between countries bordered by the Mekong River as suggested by Chewapreecha et al [25]. However, even if strong similarities exist between phylogenies based on Whole Genome Sequencing (WGS) and MLST, whole-genome phylogenetic analysis has also revealed limitations in using MLST for inferring the geographical origin of STs due to very high rates of recombination [30,31].

In terms of antibiotic susceptibility, our study indicates that *B*. *pseudomallei* isolates from Cambodia generally remain susceptible to the primary antibiotics used for treating melioidosis in the country [7,32]. With the exception of MBL17-12 (ST196), all isolates tested were susceptible to the antibiotics examined. However, even though it was susceptible, MBL17-19 showed MIC values at the upper limit for amoxicillin-clavulanic acid and ceftazidime. Reduced antibiotic susceptibility to ceftazidime and amoxicillin-clavulanic acid has been reported in Thailand [6]. This new observation in Cambodia highlights the importance of regularly monitoring antibiotic sensitivity. Additionally, MBL17-12 exhibited resistance to SXT, which functions by inhibiting the dihydrofolate reductase enzymes essential for tetrahydrofolate production in *B*. *pseudomallei*. SXT is the drug of choice for the eradication phase in Cambodia [32]. As reported by several authors, SXT resistance may be rare in *B*. *pseudomallei* but E-test gives acceptable results since they demonstrated concordance of results between broth dilution, the gold standard method and the E-test [33,34]. Our E-test results reveal a true resistance and therefore, call for further exploration of this phenotype in Cambodian *B*. *pseudomallei* strains. Resistance to SXT has been previously documented in *B*. *pseudomallei* strains in Thailand and Vietnam [6,35,36], whereas previous studies in Cambodia suggested that such resistance is rare within the country [35]. This resistance arises from various mutations, including those in *bpeT*, *bpeS*, and *folM* genes and further investigations could provide valuable insights into the genome of MBL17-12 [4,37].

To conclude, this study examined the sequence types (STs) of *B*. *pseudomallei* in Phnom Penh, Cambodia, along with their resistance profiles. MLST analysis of *B*. *pseudomallei* clinical isolates from Cambodia reveals a close association with Southeast Asian isolates, particularly those from Thailand, as well as a high level of diversity. Therefore, additional genomic data are then now essential for a better understanding of *B*. *pseudomallei* transmission in Cambodia and across SEA. Additionally, a One Health approach, which includes both human, environmental (mainly soil) and animal compartments, would be essential to decipher the epidemiology of *B*. *pseudomallei* in Cambodia. Regarding Antibiotic susceptibility, the identification of resistance to SXT and reduced susceptibility to ceftazidime and amoxicillin-clavulanic acid in this study suggests the need for further exploration of the antibiotic resistance status of *B*. *pseudomallei* in Cambodia and calls for regular monitoring of antibiotic susceptibility.

## Supporting information

S1 Table MLST profiles for the 11 supplementary references. (DOCX)

S2 Table MLST profiles available on PubMLSt database for "country" = "Cambodia" (n = 179). (XLSX)
