# Supplementary material for: Multilocus sequence typing of clinical Burkholderia pseudomallei isolates from Cambodia
Source: PLoS Negl Trop Dis. 2024 Nov 14;18(11):e0012652. doi: 10.1371/journal.pntd.0012652 (PMC11594482; doi:10.1371/journal.pntd.0012652)
Supplement: S1 Table — (DOCX) [file pntd.0012652.s001.docx]

**S1 Table. MLST profiles for the 11 supplementary references**

**(doc)**

| **ST** | **year** | **ace** | **gltB** | **gmhD** | **lepA** | **lipA** | **narK** | **ndh** | **ST geographical distribution***  **(number of isolates)** | **Comment** |
| --- | --- | --- | --- | --- | --- | --- | --- | --- | --- | --- |
|  |  |  |  |  |  |  |  |  |  |  |
| 203 | 2001 | ***4*** | 4 | 3 | 3 | 1 | 4 | 1 | TH (2), LA (1) | Close to ST1858 |
| 55 | 2017 | 3 | ***1*** | 3 | 3 | 1 | 4 | 1 | CN (16), MY (2) | Close to ST1858 |
| 65 | 1984 | 3 | 4 | 3 | 3 | 1 | ***3*** | 1 | TH (1) | Close to ST1858 |
| 66 | 1990 | 3 | 4 | 3 | ***4*** | 1 | 4 | 1 | TH (1) | Close to ST1858 |
| 705 | 1989 | 3 | 4 | ***2*** | 3 | 1 | 4 | 1 | CN (1) | Close to ST1858 |
| 821 | MD | 3 | 4 | 3 | 3 | 1 | 4 | ***6*** | VN (1) | Close to ST1858 |
| 960 | 2015 | 1 | 2 | 3 | 2 | 1 | ***1*** | 3 | IN (3), US (1) | Close to ST2065 |
| 346 | 2018 | 1 | 2 | 3 | 2 | 1 | 4 | ***1*** | MM (1), FR (1) | Close to ST2065 |
| 1730 | 2019 | 4 | 1 | 3 | 2 | 1 | 1 | 1 | SF (1) | Close to ST2066 |
| 243 | 1994 | 1 | 2 | 13 | 4 | 15 | 12 | 1 | AU (14) | outgroup |
| 999 | 2011 | 2 | 50 | 13 | 2 | 2 | 2 | 1 | AU (1) | outgroup |

*From pubMLST database.

Abbreviations : AU, Australia, CN, China ; FR, France ; IN, India ; LA, Lao People's Democratic Republic ; MM, Myanmar ; MY, Malaysia ; MD, missing data ; SF, South Africa ; ST, Sequence Type ; TH, Thailand ; US, United States of America ; VN, Vietnam.

Alleles in bold and italics differ from the alleles described for the novel ST 1858, ST2065 and St 2066
